# Proficiency in Robotic Cardiac Surgery: Toward a Brazilian Training Curriculum

**DOI:** 10.21470/1678-9741-2026-0016

**Published:** 2026-09-01

**Authors:** Robinson Poffo, Sergio Augusto Fudaba Curcio, Anderson da Rosa Rosado, Stephanie Senna, Renato Bastos Pope, Alisson Parrilha Toschi

**Affiliations:** 1 Department of Cardiovascular Surgery, Einstein Hospital Israelita, São Paulo, São Paulo, Brazil; 2 Hospital Regional Hans Dieter Schmidt, Palhoça, Santa Catarina, Brazil; 3 Department of Cardiovascular Surgery, Hospital Santa Casa de Curitiba, Curitiba, Paraná, Brazil

**Keywords:** Robotic Surgical Procedures, Cardiac Surgery, Surgical Training, Simulation Training, Competency-Based Education.

## Abstract

**Introduction:**

Robotic cardiac surgery has evolved significantly over the past decades, offering enhanced precision, improved visualisation, and minimally invasive approaches. Despite its global expansion, its adoption in Brazil remains limited, particularly due to the absence of a structured and specialty-specific training pathway.

**Objective:**

To present a structured proposal for the implementation of robotic cardiac surgery training in Brazil, based on current national regulations and international experience.

**Methods:**

This study consists of a narrative review combined with a policy analysis of Brazilian regulatory documents and international training models. Evidence regarding simulation-based training, structured curricula, and competency-based progression was analysed to support the proposed framework.

**Results:**

Current Brazilian regulations provide general guidance for robotic surgery but lack specific criteria for cardiac surgery training. In contrast, international models emphasise structured progression, simulation-based training, mentorship, and outcome-based evaluation. Based on these findings, a hybrid training model is proposed, combining short-term certification programmes and dedicated fellowship pathways.

**Conclusion:**

The implementation of a structured and standardised training pathway is essential for the safe expansion of robotic cardiac surgery in Brazil. Aligning national policies with international best practices may facilitate broader adoption while ensuring surgical proficiency and patient safety.

## INTRODUCTION

**Table:** 

Abbreviations, Acronyms & Symbols			
**AATS**	**= American Association for Thoracic Surgery**	**LUMC**	**= Leiden University Medical Center**
**AMB**	**= Associação Médica Brasileira**	**MICS**	**= Minimally invasive cardiac surgery**
**CABG**	**= Coronary artery bypass grafting**	**MVR**	**= Mitral valve repair/replacement**
**CBC**	**= Colégio Brasileiro de Cirurgiões**	**RQE**	**= Registro de Qualificação de Especialista**
**CFM**	**= Conselho Federal de Medicina**	**STS**	**= Society of Thoracic Surgeons**
**dVSS**	**= da Vinci Skills Simulator**	**TTP**	**= Technology Training Pathway**
**EACTS**	**= European Association for Cardio-Thoracic Surgery**	**USA**	**= United States of America**
**FRS**	**= Fundamentals of Robotic Surgery**	**VR**	**= Virtual reality**
**GEARS**	**= Global Evaluative Assessment of Robotic Skills**		

Robotic cardiac surgery represents a significant advancement in cardiovascular surgery, enabling improved precision, enhanced visualisation, and minimally invasive approaches. Since its initial clinical application in the late 1990s, the field has evolved substantially, following pioneering work by Alain Carpentier and Friedrich Wilhelm Mohr, who independently performed early robot-assisted mitral valve procedures that established the feasibility of this technology^[^1^,^2^]^.

Since then, continuous technological refinement and growing clinical experience have expanded the application of robotic platforms across a wide range of cardiac procedures, particularly in high-volume centres in North America and Europe^[^3^]^.

In Brazil, robotic cardiac surgery was introduced in 2010, with the initial experience reported by Poffo et al.^[^4^-^6^]^, demonstrating its feasibility, safety, and reproducibility in different cardiac procedures.

Despite these advances, the adoption of robotic cardiac surgery in Brazil remains limited. Contributing factors include restricted availability of robotic platforms, variability in institutional implementation, and, importantly, the lack of a structured and standardised training pathway specific to cardiac surgery.

The increasing use of robotic systems has raised concerns regarding the learning curve and its potential impact on patient safety. Consequently, structured training programmes have been developed in several countries to ensure adequate technical proficiency prior to independent clinical practice. However, in Brazil, the implementation of such structured, specialty-specific training pathways remains limited^[^7^,^8^]^.

In this context, this study aims to present potential strategies to support the development and advancement of robotic cardiac surgery training in Brazil through a structured proposal grounded in current national regulations and international experience. It further emphasises the importance of collaboration among academic institutions, physicians, healthcare organisations, and industry in establishing efficient and accessible training pathways. The conceptual framework proposed in this study is summarised in the Central Illustration.

**Figure:**
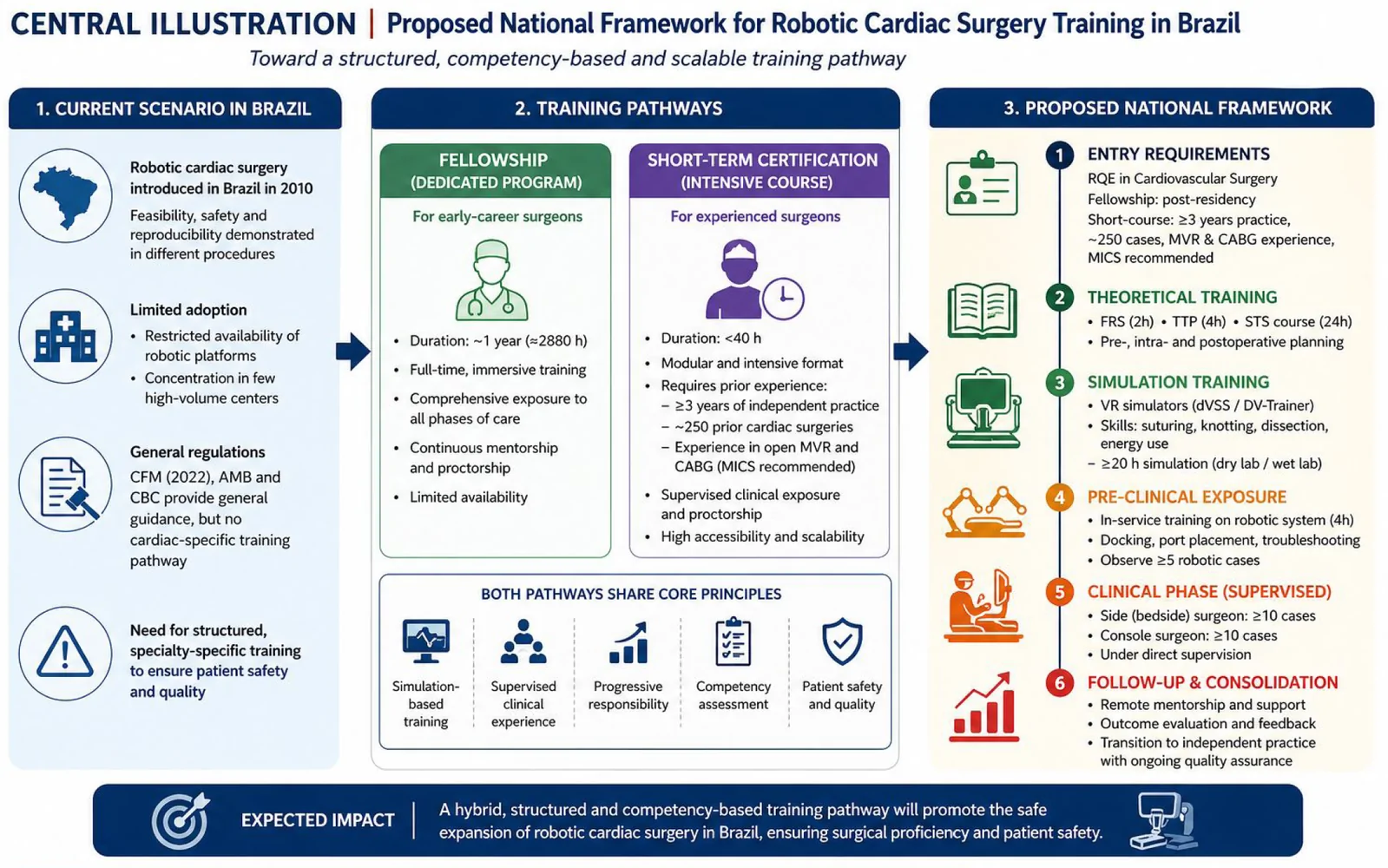
**Central Illustration** - *Proposed national framework for robotic cardiac surgery training in Brazil. This illustration summarises the current scenario, existing training pathways, and a proposed structured, competency-based model for robotic cardiac surgery training. The model integrates two complementary pathways - fellowship and short-term certification - and outlines a stepwise progression including entry requirements, theoretical instruction, simulation-based training, supervised clinical practice, and structured follow-up. This hybrid approach aims to promote safe implementation, scalability, and standardisation of robotic cardiac surgery training. AMB=Associação Médica Brasileira; CABG=coronary artery bypass grafting; CBC=Colégio Brasileiro de Cirurgiões; CFM=Conselho Federal de Medicina; dVSS=da Vinci Skills Simula-tor; FRS=Fundamentals of Robotic Surgery; MICS=minimally invasive cardiac surgery; MVR=mitral valve repair/replacement; RQE=Registro de Qualificação de Especialista; STS=Society of Thoracic Surgeons; TTP=Technology Training Pathway; VR=virtual reality.*

## METHODS

This study was designed as a narrative review combined with a policy analysis. Brazilian regulatory documents were reviewed, including guidelines from the Conselho Federal de Medicina (CFM) and national surgical organisations such as the Associação Médica Brasileira (AMB) and the Colégio Brasileiro de Cirurgiões (CBC)^[^9^]^.

In parallel, a targeted literature review was conducted focusing on robotic surgery training models, simulation-based education, and structured curricula, including randomized trials, consensus statements, and multicentre validation studies^[^10^-^12^]^.

International training frameworks from North America, Europe, and Asia were analysed to identify common structural elements, competency benchmarks, and implementation strategies^[^8^,^12^]^.

Based on these findings, a structured training proposal applicable to the Brazilian context was developed.

## RESULTS

The analysis of the available literature and regulatory frameworks revealed a heterogeneous landscape of robotic surgery training, with significant variability across countries, institutions, and surgical specialties. Despite these differences, common foundational principles were consistently identified, including patient safety, progressive acquisition of technical skills, simulation-based training, supervised clinical exposure, and structured assessment of proficiency.

In Brazil, current regulations provide a general framework for robotic surgery certification and institutional requirements. However, no dedicated, specialty-specific pathway for robotic cardiac surgery has been formally established. In contrast, international models - particularly those developed in North America and Europe - have incorporated structured curricula, mentorship, proctoring, and competency-based progression.

Based on these findings, the following sections summarise the current regulatory landscape, the evidence supporting structured training, and international experience, culminating in a proposed training model adapted to the Brazilian context.

### Brazilian Regulatory Framework

In Brazil, robotic surgery is regulated by the CFM, which classifies it as a high-complexity procedure requiring appropriate institutional infrastructure, trained surgical teams, and formal credentialing prior to independent practice^[^13^]^.

Additional guidance is provided by national organisations such as the AMB and the CBC, which define general requirements for training, certification, and institutional accreditation in robotic surgery^[^9^]^.

However, these regulations are not specific to cardiac surgery and do not establish detailed criteria regarding minimum procedural volumes, objective competency assessment, or structured progression of training. As a result, there is considerable variability in how robotic surgery training is implemented across institutions in Brazil.

Certification in robotic surgery in Brazil requires two main components: specialist qualification and structured training.

### Specialist Qualification

Candidates must:

• Hold a recognised surgical specialty certification through an AMB-accredited programme or a residency recognised by the Ministério da Educação
• Possess an active specialist qualification record (Registro de Qualificação de Especialista [RQE]), in accordance with CFM regulations

### Structured Training Pathway (Model of the Colégio Brasileiro de Cirurgiões)

The training pathway proposed by the CBC is divided into basic (pre-clinical) and advanced (clinical) phases, ensuring progressive acquisition of theoretical knowledge and technical competencies.

#### Basic phase (pre-clinical)

• Theoretical training: ≥ 30 hours
• Simulation training: ≥ 20 hours
• In-service exposure: ≥ 4 hours
• Procedural observation: ≥ 5 robotic procedures This phase includes:
• Modular theoretical instruction
• Video-based learning
• Console-based simulation
• Familiarisation with robotic platforms and instrumentation

#### Advanced phase (clinical)

• Assistant (side surgeon): ≥ 10 procedures
• Console surgeon (under supervision): ≥ 10 procedures
• Final evaluation and certification by a qualified tutor

This phase represents the transition from simulation to clinical practice, with progressive increase in operative responsibility.

### Tutor Qualification

To be recognised as a robotic surgery tutor by the CBC, surgeons must:

• Hold an active RQE
• Be formally certified in robotic surgery
• Demonstrate experience in ≥ 50 complex robotic procedures

### Critical Limitations

Although this framework provides a structured pathway applicable across surgical specialties, it remains generic and not tailored to the specific demands of cardiac surgery. In particular, it does not define:

• Procedure-specific volume thresholds for robotic cardiac surgery
• Validated, objective competency assessment metrics
• Specialty-specific progression criteria

In contrast, international training frameworks incorporate structured curricula, simulation-based validation, objective performance metrics, and clearly defined competency benchmarks, often within dedicated cardiac surgery programmes (Table 1).

**Table 1:** Comparison of robotic surgery training requirements between Brazil and international frameworks.

Component	Brazil (CBC/AMB/CFM)	International (AATS/STS/EACTS)
Basic training (pre-clinical)	≥ 30 h theory + ≥ 20 h simulation	Structured modules + simulation
Observation	≥ 5 cases	Required
Advanced training (clinical)	≥ 10 assistant + ≥ 10 console	≥ 20 - 50 console cases
Competency assessment	Tutor-based	Objective metrics (*e.g.*, GEARS, FRS)
Fellowship availability	Limited	Widely available

AATS=American Association for Thoracic Surgery; AMB=Associação Médica Brasileira; CBC=Colégio Brasileiro de Cirurgiões; CFM=Conselho Federal de Medicina; EACTS=European Association for Cardio-Thoracic Surgery; FRS=Fundamentals of Robotic Surgery; GEARS=Global Evaluative Assessment of Robotic Skills; STS=Society of Thoracic Surgeons

This table summarises the key components of robotic surgery training as defined by Brazilian regulatory bodies (CFM, AMB, and CBC) and compares them with established international frameworks supported by major societies such as AATS, STS, and EACTS. Differences are highlighted in terms of training structure, simulation requirements, procedural volume, competency assessment methods, and availability of fellowship programmes.

### National Context and Development of Robotic Cardiac Surgery in Brazil

In the absence of a formal national training pathway, early adoption of robotic cardiac surgery in Brazil relied on surgeons trained abroad. Subsequently, only a limited number of centres established structured programmes.

Two main centres currently represent reference institutions:

• Instituto Poffo de Cirurgia Cardiovascular (São Paulo, since 2010)
• Uberlândia Medical Center (Minas Gerais, since 2019)

These centres accumulated significant experience and became responsible for the development of local training initiatives, including mentorship and tutor certification within the CBC framework.

Surgeons from these institutions, based on surgical volume and expertise, achieved certification as robotic surgery tutors (≥ 50 complex procedures), enabling dissemination of training and expansion of robotic programmes nationally.

### Robotic Cardiac Surgery Developed During Medical Residency

The integration of robotic cardiac surgery into cardiovascular surgery residency programmes represents a fundamental milestone for the educational advancement of the specialty, particularly when considering long-term outcomes. Training in this area during residency provides future surgeons with valuable exposure to advanced technologies and minimally invasive approaches^[^14^,^15^]^.

This early exposure is especially relevant in regions where access to robotic technology is limited, as it allows residents to become familiar with evolving surgical techniques and aligns with global trends in surgical education^[^14^,^15^]^.

However, despite more than two decades of technological development, structured exposure to robotic cardiac surgery during residency remains limited in Brazil. This limitation reflects the scarcity of specialised centres capable of offering such training, particularly because many major training institutions do not yet incorporate robotic cardiac surgery into their clinical practice.

### Short-Term Certification Courses (Intensive Pathway)

Short-term certification courses, typically lasting < 40 hours, provide an initial introduction to robotic surgery.

Historically, training has been predominantly supported by industry-led programmes, particularly those developed by Intuitive Surgical®, through a pathway including^[^16^]^:

• Basic phase (pre-clinical):
  • Fundamentals of Robotic Surgery (FRS)
  • Technology Training Pathway (TTP)
  • Simulation training (≥ 20 hours)
  • Theoretical training (≥ 30 hours)
  • Observation of ≥ 5 procedures
• Advanced phase (clinical):
  • Assistant in ≥ 10 procedures
  • Console surgeon in ≥ 10 procedures
  • Final supervised evaluation

Simulation plays a central role in this process, with validated platforms such as the da Vinci Skills Simulator (dVSS) and DV-Trainer (Mimic Technologies) providing objective performance metrics that enable standardisation of skills acquisition and support progressive learning^[^16^]^.

More recently, Brazilian organisations - including the AMB, CFM, and CBC - have sought to standardise certification pathways across specialties^[^13^]^. In this context, structured models have already been implemented in other fields, such as thoracic surgery, where formalised training requirements include defined theoretical, simulation-based, and supervised clinical components^[^17^]^.

This transition towards society-based certification models is particularly relevant in the context of emerging robotic platforms, as it promotes standardisation and facilitates surgeon adaptability across different systems. At present, however, a structured and specialty-specific national framework for robotic cardiac surgery has not yet been formally established.

### Robotic Cardiac Surgery Fellowship

Another established pathway for training in robotic cardiac surgery is the fellowship model, widely adopted internationally. In 2023, the Instituto Poffo de Cirurgia Cardiovascular (São Paulo, Brazil) launched the first fellowship in robotic and minimally invasive cardiovascular surgery in Latin America, consisting of a one-year, full-time structured programme conducted in high-standard centres^[^18^]^.

Training encompasses the full continuum of care (preoperative, intraoperative, and postoperative) and is complemented by theoretical instruction, case discussions, and research activities. Technical progression follows a staged model, from observation to side-surgeon participation and, ultimately, supervised console experience, with fellows expected to perform at least ten procedures under proctorship^[^18^]^.

This pathway, typically undertaken after residency, corresponds to approximately 2,880 hours of dedicated training and represents a comprehensive model for developing robotic cardiac surgeons, ensuring both technical proficiency and patient safety. It aligns with international recommendations emphasising structured, team-based training and competency-based progression^[^18^,^19^]^. Despite these advantages, the requirement for full-time dedication and the limited availability of positions restrict its accessibility, particularly for experienced surgeons.

Internationally, fellowship-based training is integrated into structured educational frameworks supported by major societies and high-volume institutions. Programmes associated with organisations such as the American Association for Thoracic Surgery (AATS), Society of Thoracic Surgeons (STS), and European Association for Cardio-Thoracic Surgery, as well as centres including Cleveland Clinic, Mayo Clinic, Herzzentrum Leipzig, and Leiden University Medical Center, incorporate simulation-based training, mentorship, and progressive operative responsibility within standardised curricula and competency-based assessment models (Table 2).

**Table 2:** Examples of international robotic cardiac surgery training programmes and institutional models.

Institution / Society	Country	Model	Key Features	Official Source
AATS	USA	Fellowship / grants	Structured robotic training, mentorship, funded fellowships	https://www.aats.org
STS	USA	Courses / workshops	Simulation-based training, clinical integration	https://www.sts.org
EACTS	Europe	Courses / certification	Structured training programmes, competency-based education	https://www.eacts.org
Cleveland Clinic	USA	Institutional Program	High-volume robotic cardiac surgery training	https://my.clevelandclinic.org
Mayo Clinic	USA	Fellowship	Advanced robotic cardiac surgery fellowship	https://www.mayoclinic.org
Herzzentrum Leipzig	Germany	Academic Program	Pioneer centre in robotic and minimally invasive cardiac surgery	https://www.herzzentrum-leipzig.de
LUMC	Netherlands	Academic Program	Structured robotic and minimally invasive training	https://www.lumc.nl
Intuitive Surgical (da Vinci Training)	Global	Industry-supported	Simulation modules, stepwise certification pathway	https://www.intuitive.com/en-us/education

AATS=American Association for Thoracic Surgery; EACTS=European Association for Cardio-Thoracic Surgery; LUMC=Leiden University Medical Center; STS=Society of Thoracic Surgeons; USA=United States of America

This table presents representative examples of robotic cardiac surgery training programmes from major international societies (AATS, STS, and EACTS) and high-volume academic centres. It outlines the type of training model, key structural components, and institutional characteristics that contribute to the development of structured, competency-based robotic surgery training pathways.

### Certification Abroad

International certification pathways, particularly those supported by the AATS, provide a structured and competency-based framework for robotic cardiac surgery training^[^7^]^. These programmes require staged progression, including formal coursework, simulation-based training, supervised clinical experience, and longitudinal mentorship.

Candidates are typically expected to meet strict prerequisites, including substantial experience in cardiac surgery, such as a minimum of 250 coronary artery bypass grafting procedures or 75 mitral valve surgeries with documented outcomes, as well as prior exposure to minimally invasive techniques^[^20^]^. In addition, completion of at least 50 robotic cases under supervision, with submission of outcomes and follow-up data, is required for certification^[^7^]^.

Training is further supported by standardised industry-aligned curricula, such as the Intuitive Surgical Pathway (TR100 - TR500), which integrates simulation, procedural training, and progressive technical refinement. These programmes are usually conducted in high-volume centres with dedicated multidisciplinary teams and institutional requirements, including minimum annual surgical volumes and specialised perioperative support^[^20^]^.

## DISCUSSION

Certification in robotic cardiac surgery is a structured and competency-driven process designed to ensure safe implementation of a highly complex surgical technology. Two main training pathways are consistently recognised worldwide: short-term structured certification programmes and dedicated fellowship training. Although distinct in format and duration, both pathways share core principles, including simulation-based skill acquisition, supervised clinical exposure, and progressive responsibility, as illustrated in Figure 1.

**Fig. 1:**
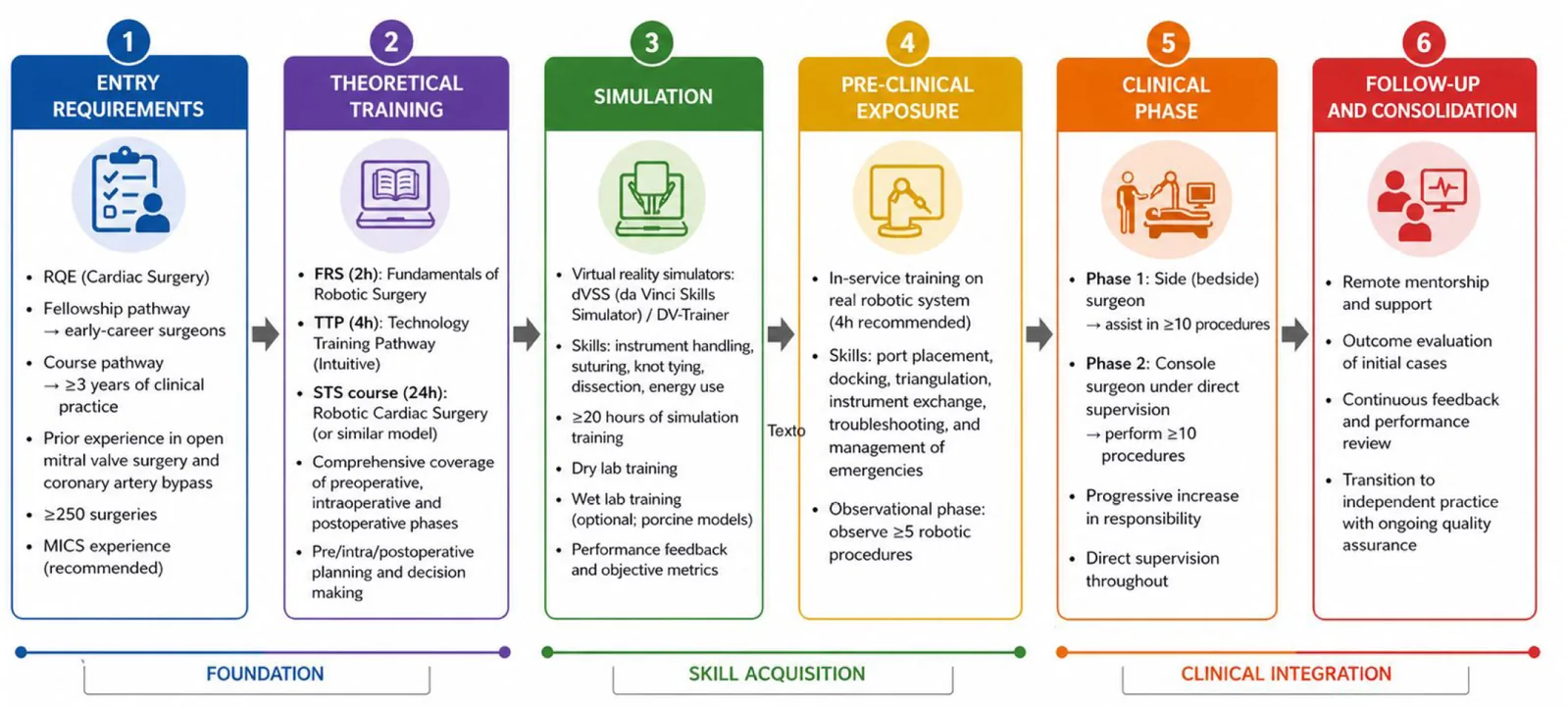
Proposed structured training pathway for robotic cardiac surgery. This schematic summarises a stepwise, competency-based training model integrating entry requirements, theoretical instruction, simulation-based training, pre-clinical exposure, supervised clinical practice, and structured follow-up. The model is based on current literature and institutional training frameworks, emphasising progressive skill acquisition and patient safety. MICS=minimally invasive cardiac surgery; RQE=Registro de Qualificação de Especialista; STS=Society of Thoracic Surgeons.

The fellowship model is often regarded as the most comprehensive training pathway due to its immersive nature and extended duration. Typically conducted over one year, with approximately 2,880 hours of dedicated training, this approach provides continuous exposure to all phases of patient care, including preoperative assessment, intraoperative management, and postoperative follow-up. This structure aligns with contemporary international recommendations, which emphasise team-based training, longitudinal mentorship, and progressive competency development^[^8^]^.

In addition to technical skill acquisition, fellowship programmes promote a broader understanding of robotic cardiac surgery, including decision-making, complication management, and multidisciplinary coordination. These elements are critical, as robotic surgery introduces a distinct set of psychomotor and cognitive challenges that are not fully transferable from conventional or laparoscopic techniques^[^21^]^.

However, despite its educational advantages, the fellowship pathway presents important limitations. The requirement for full-time dedication restricts accessibility, particularly for established surgeons, and the limited availability of structured programmes does not meet current demand. This constraint has been consistently reported across different healthcare systems and reflects the concentration of robotic surgery in high-volume centres.

Conversely, short-term certification programmes provide a more accessible and scalable alternative, particularly for surgeons seeking to incorporate robotic techniques into their practice without interruption of clinical activity. When structured appropriately, these programmes integrate theoretical instruction, simulation-based training, and supervised clinical experience. Evidence from simulation-based studies demonstrates that structured training significantly improves technical performance and accelerates skill acquisition, supporting its role as a fundamental component of robotic training^[^10^]^. A comparative overview of these two training pathways is presented in Table 3 and Figure 2.

**Table 3:** Comparative characteristics of robotic cardiac surgery training pathways.

Feature	Fellowship	Short-term certification
Duration	~ 1 year (≈ 2880 h)	< 40 h
Target profile	Early-career surgeons	Experienced surgeons
Entry requirements	Post-residency	≥ 3 years practice, ~ 250 cases
Training model	Full immersion	Modular / intensive
Simulation	Integrated	Essential component
Clinical exposure	Extensive	Limited
Supervision	Continuous	Procedure-based
Accessibility	Limited	High
Scalability	Low	High

This table summarises the main differences between fellowship-based and short-term certification pathways, including duration, candidate profile, training structure, clinical exposure, and scalability. The comparison highlights the complementary nature of both models in the development of robotic cardiac surgery training.

**Fig. 2:**
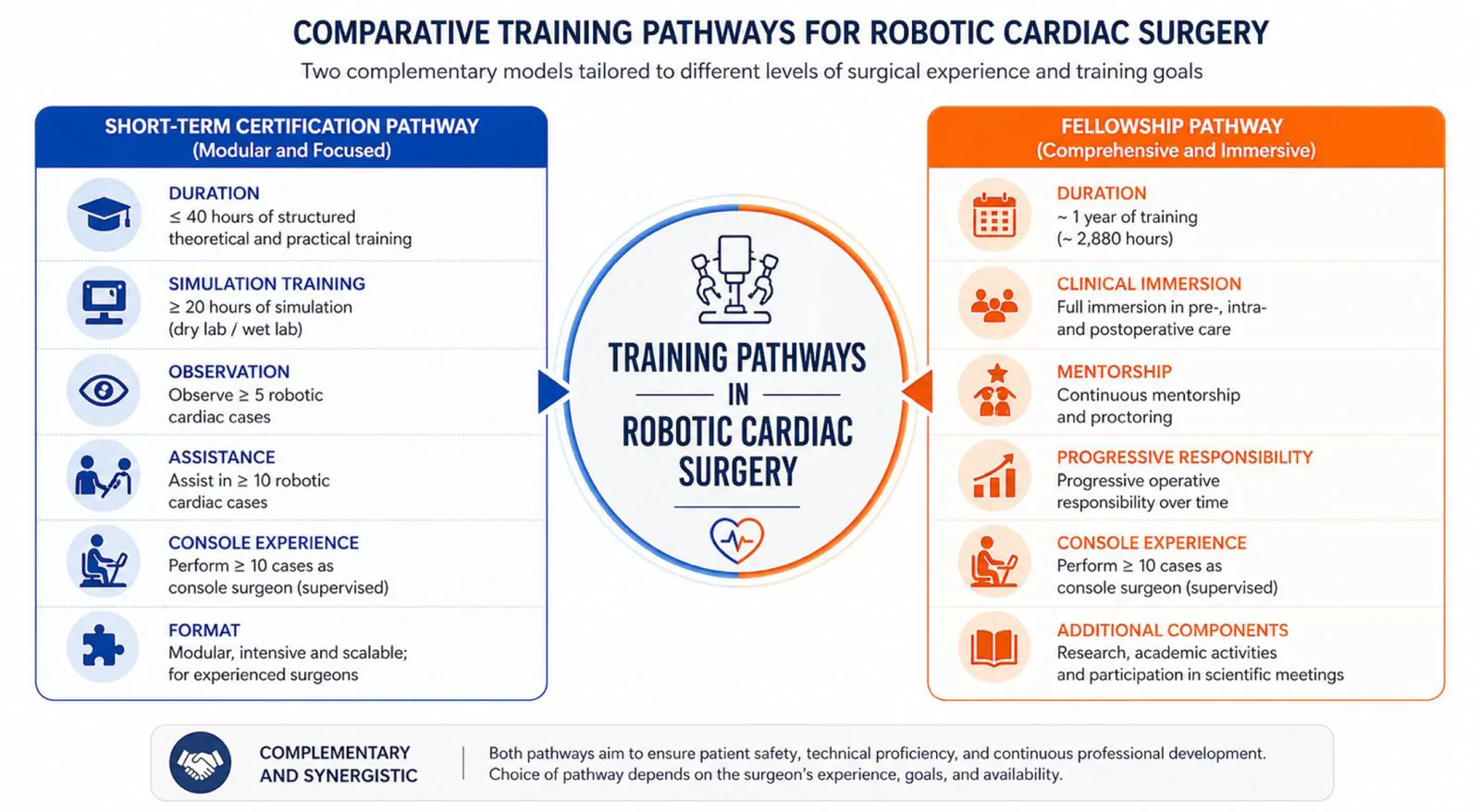
Comparative training pathways for robotic cardiac surgery. This figure illustrates the two principal training pathways currently adopted in robotic cardiac surgery: short-term certification and fellowship programmes. The short-term pathway (blue) represents a structured and modular approach focused on the acquisition of core technical competencies through theoretical instruction, simulation, and limited supervised clinical exposure. In contrast, the fellowship pathway (orange) represents a comprehensive and immersive model, characterised by extended clinical exposure, continuous mentorship, and progressive operative responsibility. These pathways are complementary and address different levels of surgical experience, supporting both accessibility and depth of training.

A structured and competency-based training pathway is essential for the safe adoption of robotic cardiac surgery. Both fellowship programmes and short-term certification courses should require baseline qualification, including formal certification in cardiovascular surgery and an active RQE. While fellowship programmes are primarily intended for early-career surgeons entering directly after residency, short-term certification pathways are more appropriate for experienced surgeons and should ideally require at least three years of independent clinical practice, prior experience in open mitral valve surgery and coronary artery bypass grafting, and a documented operative volume of approximately 250 procedures. Previous exposure to minimally invasive cardiac surgery may further facilitate adaptation to robotic techniques^[^7^,^8^,^20^]^. Training should follow a structured, stepwise progression integrating theoretical instruction, simulation, and supervised clinical practice. Foundational curricula such as the FRS and the TTP provide the initial cognitive framework, followed by dedicated courses (*e.g.*, STS-based models) covering preoperative, intraoperative, and postoperative management. Simulation-based training - including virtual reality platforms (dVSS, DV-Trainer), dry lab, and wet lab models - plays a central role in developing psychomotor skills, with a recommended minimum of 20 hours of simulation. This is complemented by in-service pre-clinical training and structured observational exposure to at least five robotic procedures. The clinical phase involves progressive responsibility, including participation as a bedside surgeon in approximately ten procedures, followed by supervised console experience in at least ten cases. Finally, structured follow-up of initial cases, often supported by mentorship, is recommended to ensure safe transition to independent practice and maintenance of surgical quality^[^10^,^16^,^21^-^23^]^.

The importance of simulation is further reinforced by the unique characteristics of robotic surgery, which requires adaptation to console-based operation, altered ergonomics, and limited haptic feedback. Simulation allows skill development in a controlled environment, reducing the learning curve and minimising patient risk^[^21^]^. In addition, validated curricula such as the FRS have demonstrated improved performance and reduced technical errors in controlled settings^[^11^]^.

Despite these advances, a consistent finding across the literature is the lack of standardised, universally adopted training curricula. Even internationally, training pathways remain heterogeneous, with variability in duration, assessment methods, and credentialing criteria^[^22^]^. In Brazil, although regulatory frameworks established by the CFM and national societies define general requirements for robotic surgery, these remain non-specific to cardiac surgery and do not include detailed competency metrics or procedural thresholds^[^13^]^.

Taken together, these findings support a hybrid training strategy, integrating the strengths of both pathways. Fellowship programmes provide depth and comprehensive training, while short-term certification offers scalability and accessibility. The combination of these approaches may represent the most effective strategy for expanding robotic cardiac surgery while maintaining high standards of training and patient safety.

### Limitations

As a narrative review and policy analysis, this study is influenced by the heterogeneity of available training frameworks and differences in regulatory and institutional contexts across countries. Therefore, elements derived from international models may require adaptation to the Brazilian healthcare and training environment. The proposed framework should be regarded as a foundation for future development as national experience and specialty-specific training standards evolve.

## CONCLUSION

Robotic cardiac surgery has demonstrated feasibility, safety, and favourable clinical outcomes in selected centres, both internationally and in Brazil^[^4^-^6^]^. However, its broader implementation remains closely linked to the availability of structured and standardised training pathways.

Current evidence supports the need for competency-based training models that incorporate simulation, supervised clinical experience, and progressive responsibility. While international programmes have advanced toward structured and validated curricula, the Brazilian framework remains general and not specific to cardiac surgery.

The coexistence of two main training pathways - short-term certification programmes and fellowship-based training - offers a practical foundation for developing a national strategy. A hybrid model, integrating accessibility with depth of training, may facilitate the safe and scalable expansion of robotic cardiac surgery in Brazil. The development of coordinated efforts among academic institutions, professional societies, and training centres will be essential to establish consistent standards, improve training quality, and ensure patient safety.

## Data Availability

The authors declare that all data supporting the findings of this study are derived from publicly available sources cited within the article.
